# Psychopaths and blame: The argument from content

**DOI:** 10.1080/09515089.2012.729485

**Published:** 2013-01-15

**Authors:** Neil Levy

**Affiliations:** Head of Neuroethics at the Florey Institute of Neuroscience and Mental Health.

**Keywords:** Blame, Mental Time Travel, Moral Knowledge, Personhood, Psychopathy

## Abstract

The recent debate over the moral responsibility of psychopaths has centered on whether, or in what sense, they understand moral requirements. In this paper, I argue that even if they do understand what morality requires, the content of their actions is not of the right kind to justify full-blown blame. I advance two independent justifications of this claim. First, I argue that if the psychopath comes to know what morality requires via a route that does not involve a proper appreciation of what it means to cause another harm or distress, the content of violations of rules against harm will be of a lower grade than the content of similar actions by normal individuals. Second, I argue that in order to intend a harm to a person—that is, to intend the distinctive kind of harm that can only befall a person—it is necessary to understand what personhood is and what makes it valuable. The psychopath's deficits with regard to mental time travel ensure that s/he cannot intend this kind of harm.

## 1. Introduction

Should we blame psychopaths for their harmful actions? Much of the recent debate over this question has concerned the capacities of psychopaths, and in particular, whether they are capable of moral knowledge. Some philosophers have argued that psychopaths’ deficits in empathy cause them to have difficulties with the acquisition of moral concepts, and that these difficulties, in turn, entail that they lack moral responsibility for their actions.

This argument is controversial for a number of reasons. First, though it is uncontroversial that psychopaths have deficits in empathy, it is controversial whether these deficits cause a corresponding deficit in the acquisition of moral concepts. It may be that empathy (of the kind affected) is not involved in the normal acquisition of moral concepts; alternatively, even if the route to the acquisition of moral concepts typically involves empathy, there may be alternative routes to the acquisition of such concepts (Vargas & Nichols, 2007). Further, it is controversial whether the moral concepts in question are required for moral responsibility. A psychopath may not understand the distinctive kind of harm involved in causing someone distress, but they are entirely capable of understanding that some kind of actions are forbidden by law; this might be sufficient to attribute to them some degree of moral responsibility for their actions (Blair, 2007). Finally, we might blame them because their actions express their lack of moral concern, rather than in spite of this deficit (Scanlon, 1998). All these routes to blaming the psychopath have been defended by some philosopher or another.

In this paper, I want to defend an alternative route to excusing at least some psychopaths. Regardless of whether they are able to acquire the concept of the distinctive kind of harm involved in causing another agent distress, and regardless of whether their understanding of the law might be sufficient to compensate for any (possible) lack of moral knowledge, I will suggest that some psychopaths should be (at least) very largely excused because their actions do not have the right kind of content, where an action's content is a function of what is intended by the agent. Psychopaths do not intend the kind of harm that can befall only persons. Their actions can, at most (assuming they have a sufficient grasp of moral concepts to be able to perform actions with a moral content at all) express a content equivalent to that which we express in harming an animal that is not a person. Indeed, I shall claim, psychopaths may be said to lack the full-blown concept of a person required for them to intend a harm to a person. They can, that is, intend a harm, and the target of that harm may be a person, but they cannot intend < a harm to a person>: personhood can neither figure in the content of their intention nor constitute a reason, for them, against their actions. This fact, I shall claim, very significantly reduces the amount of blame that can rightly be attributed to them when they do harm persons.

Prior to defending this claim, I will sketch some of the landscape of the debate over psychopathy. Independent of the argument I shall advance, there are already plausible grounds for excusing the psychopath. Though some people are unconvinced by the arguments I shall sketch and extend this in the next section, the combined force of these arguments plus the independent argument I shall subsequently advance ought to constitute a powerful case for excusing the psychopath from a great deal of the blame due to less impaired offenders.

## 2. The Moral/Conventional Distinction

A popular, but highly controversial, account of the moral capacities of psychopaths builds on work on the moral/conventional distinction task (MCT). The task, originally used to test the moral competence of children, tests to see whether subjects regard a set of wrongful acts as authority-independent. A transgression is authority-dependent just in case its wrongness depends on whether some authority has the power to allow or disallow it; otherwise it is authority-independent. Children as young as three reliably classify some transgressions into each category (Nucci, 1989; Turiel, 1977, 1983). Thus, they agree that wearing gender inappropriate clothing or talking in class is acceptable if the teacher allows it, but they deny that hitting or pulling hair is acceptable even if the teacher says it is acceptable.

There is some evidence that psychopaths fail to be able reliably to classify transgressions into those that are moral and those that are conventional (Blair, 1995, 1997). Rather, in Blair's small sample, they overclassified transgressions as authority-independent; that is, they tended to deny that any transgression is acceptable, no matter who says otherwise. This apparent deficit has been explained as the consequence of a more basic deficit that psychopaths exhibit: a deficit in empathy. Because they fail to empathize with those who suffer harms, they fail to see that this suffering constitutes an authority-independent reason against inflicting harm.^1^ Instead, they see all reasons for action as on a par. There is no more reason to refrain from causing suffering than there is to refrain from parking on a double yellow line. Both actions are against the rules, and that's the only reason for refraining from them.

On the basis of this data and convergent evidence from social psychology and evolutionary biology, some have concluded that psychopaths are blind to moral demands qua moral, and therefore should be excused from moral responsibility (Levy, 2007, 2010). According to these philosophers, since psychopaths do not understand the distinctive character of moral demands, they are not blameworthy for violating these demands. Indeed, it has been suggested that they are not even due the kind of blame that is often appropriate when ordinary agents violate conventional norms. Conventional norms, it is claimed, are often backed up by second-order moral norms: we ought not to jump the queue (say) not only because queue-jumping is against the rules, but because other agents have a reasonable expectation that the rule will be obeyed and structure their behavior with this expectation in mind. Given the role that conventions play in our lives, violating such rules tends to interfere with people's plans and policies, thereby impairing their ability to shape their lives as they choose (at least in the short term), and quite possibly causing them distress. It seems plausible to think that an inability to appreciate the distinctive force of moral norms therefore reduces the power of merely conventional norms. If this is correct, the psychopath is due only the blame that attaches to the violation of the most trivial conventions (those the violation of which do not inconvenience others or cause them distress; perhaps the rules of fashion might be an example).

However, every step of this argument has come in for criticism. First, it may be doubted whether the data is sufficiently robust to support the argument. Attempts at replication have provided only partial support for Blair's findings. Utilizing the youth version of the revised psychopathy checklist, Dolan and Fullam (2010) found that subjects high in psychopathy could distinguish moral from conventional transgressions semantically, though the subjects in their sample could not reliably use authority-independence as a criterion for morality, indicating that there are alternative routes to the acquisition of knowledge about morality (whether this ought to be counted as moral knowledge, rather than knowledge *about* morality, is unclear).

Further, the results reported by Blair are somewhat mysterious. If psychopaths do not understand the authority-independence of moral norms, why do they not claim that moral and conventional norms are alike authority-dependent? They actually do the opposite, claiming that both kinds of norms are authority-independent. Blair argues that this response is a product of the fact that his (incarcerated) sample had an incentive to show that they were reformed, to increase their chances of early release; their claim that all rules should be respected no matter what was a show designed to demonstrate their readiness to return to society. But the evidence might be taken as showing not that psychopaths think that all norms are merely conventional: rather, it might show that to them, all norms are moral (Haji, 2010; Vargas & Nichols, 2007).

Moreover, even if we accept that psychopaths’ deficits with regard to empathy leave them unable to grasp that suffering constitutes an authority-independent reason against moral harms, they may be able to grasp moral norms by an alternative route (Vargas & Nichols, 2007). Morality is not co-extensive with the domain of actions that cause suffering to victims. Some moral norms may be grounded in virtues, for instance, or (for that matter) in a system of rules—divine command theorists maintain as much.

Further, it may be held that even if psychopaths do not understand moral norms qua moral, such understanding is not necessary for moral responsibility: understanding that an action is against the rules may be sufficient for responsibility (Blair, 2007; Vargas & Nichols, 2007). Alternatively, moral responsibility might be grounded in the attitudes expressed by actions (Arpaly, 2002; Scanlon, 2008). In harming others, psychopaths evince a complete disregard for others and for their interests; this fact might be sufficient to justify blaming them.

It is theories of this last kind upon which I want to focus here. If we ground responsibility in the manner suggested, on the attitudes expressed by actions, or even if we understand the degree of blameworthiness of an agent to be a function, perhaps among other things, of the content of their attitudes, we are provided with an alternative route to mitigation, I shall suggest. I will argue that the psychopath does not express the kind of attitudes that would justify full-blown moral responsibility for his/her actions, regardless of whether s/he has acquired moral concepts.

I shall focus, first, on what we can learn from the MCT about the content of the attitude expressed by psychopaths’ harmful action. I shall offer a somewhat deflationary explanation of performance on the MCT; an explanation that acknowledges its inadequacy as a test for moral capacity tout court, and yet which makes plausible the claim that in harming others, the psychopath does not express ill will of the kind that would be in play were s/he to understand harms as we do. I will then turn to an independent argument for the claim that his/her actions do not express ill will of the kind that can only be intended with regard to persons, an argument that turns on the difficulties that psychopaths have with mental time travel.

## 3. Explaining Psychopaths’ Performance on the MCT

The MCT classifies rules as “moral” or “conventional” by reference to three criteria: authority-independence (the force of moral rules cannot be waived by authorities); seriousness (transgressions of moral rules are especially serious); and the justifications cited (moral rules are justified by reference to the welfare of victims). Proponents of the task seemed to have thought that these criteria would hang together. This assumption has come in for a great deal of criticism, empirically inspired and conceptual (Shoemaker, 2011). Rules may be justified by reference to the welfare of victims and yet not be especially serious (pinching someone hurts yet is very plausibly less seriously wrong than, say, parking across the entry ramp to the freeway at rush hour). Rules may plausibly be waived by authorities and yet clearly moral (boxers waive their right not to be punched). In the literature on psychopathy, there seems to be an assumption that authority-independence is the heart of morality, but this assumption begs the question against various views of morality, both those that make authority essential to morality (divine command theories, and cultural relativism) and those that develop basic criteria independent of authority (such as virtue ethics). Given these problems, the claim that the MCT gives us a test for morality as such must be abandoned.

In this light, Vargas and Nichols’ (2007) claim—that even if psychopaths cannot appreciate welfare-based reasons for refraining from transgressing the moral rules, there are alternative routes to the acquisition of moral knowledge—looks all the stronger. Moreover, the empirical evidence seems to back them up. Available data is difficult to interpret, but it seems clear that in some domains, at least, people high in psychopathy seem to have no problems in identifying moral rules. As already mentioned, Dolan and Fullam (2010) found that psychopaths seem to acquire knowledge of which transgressions are moral despite their difficulties with authority-independence as a criterion for morality.

Similarly, Aharoni, Sinnott-Armstrong, and Kiehl (2012) found that in a forced choice situation, psychopaths did not differ from normal controls in their ability to classify offenses into those which are authority-dependent and independent. Further, studies utilizing Haidt's five psychological foundations of morality (Haidt & Joseph, 2004) have found that though psychopaths score lower on some dimensions of moral knowledge (harm and fairness), they score normally or nearly normally on the authority, loyalty, and purity dimensions (Aharoni et al., 2012; Glenn, Iyer, Graham, Koleva, & Haidt, 2009).^2^

In older literature—prior to the current obsession with empirical data—moral philosophers often discussed the *amoralist*: the person who knew the moral rules but did not subscribe to them, perhaps using them in an inverted commas manner (see Brink, 1989, for a representative discussion). The evidence cited above is persuasive in showing that the psychopath can classify rules into authority-dependent and independent, but is consistent with the psychopath being an amoralist; with (for instance) his/her tracking the distinction but not properly appreciating the reasons that make a rule moral. In fact, this is a reasonable interpretation of the evidence. In the remainder of this section, I will defend this claim. I thereby aim to add to the case that psychopaths lack moral knowledge, and therefore ought to be excused on this basis. In subsequent sections, I will buttress the case for excusing the psychopath by arguing that whether or not the psychopath possesses moral knowledge, his/her actions lack the kind of content that justifies (full-blown) blame.

Consider Aharoni et al. (2012). As noted above, participants in Blair's original (1995) study overclassified rules as authority-independent, rather than conventional. Blair explained this behavior by reference to the fact that his sample was incarcerated: they aimed to show how thoroughly reformed and therefore ready for release they were by showing how seriously they took *all* rules, moral and conventional. In order to eliminate this incentive to impression management, Aharoni et al. used a forced response paradigm. They gave their subjects a list of transgressions and told them that half of them were rule-independently wrong. The incentive to appear to be especially law-abiding by rating all rules as seriously wrong was thereby removed. This had the effect of eliminating any effect of psychopathy on classification reliability. The authors therefore conclude that psychopaths have intact moral knowledge.

However, this is too quick. As a side-effect of eliminating the incentive to impression management, Aharoni et al. have altered the nature of the task. Blair asked his subjects to judge whether an action was wrong independently of the rules; Ahoroni et al. asked their subjects to judge whether normal individuals would think an action was wrong independently of the rules. In other words, the manipulation switched the task from a test of subjects’ own views to a test of their predictions regarding most people's views. Performing this third-person task requires not moral knowledge, but knowledge of how moral language is used. These are clearly different sets of knowledge, as can easily be seen by thinking about the dissociation between an anthropologist's moral beliefs and their beliefs about how moral language is used in a particular culture.

Further, the paradigm also had the effect of making the task easier by informing subjects that exactly half of the items belong in each category. The forced-choice format serves as a cognitive scaffold for the psychopath by providing guidance as to how many rules belong in each category. Aharoni et al. found that intelligence explained 54% of the variance in subjects’ responses; this is unsurprising since intelligence can allow agents to latch onto the patterns in how others use language. It is also worth noting that while psychopathy did not predict worse performance at the MCT, the affective facet of the psychopathy checklist (PCL-R) did predict reduced performance at the task. If, as Blair claimed, it is lack of empathy that explains deficits in the MCT, this is also unsurprising: when more cognitive strategies fail, subjects are forced to use their own judgment in assigning transgressions to moral and conventional categories, and their affective deficits would interfere with their performance.

When we consider the role of authority-independence in classification—the facts which it tracks—neither the deficits of psychopaths with regard to classification using this criterion nor their ability to classify transgressions relatively accurately is surprising. A certain kind of authority-independence obtains with regard to all supervening concepts: concepts that apply in virtue of the facts in its supervenience base being instantiated. All supervening concepts are authority-independent in the following way: they apply or fail to apply in virtue of their supervenience base, regardless of what anyone says (unless what they say features in the supervenience base of the concept). The moral transgressions upon which the MCT focus are independent of authority (that is, of the authority of non-victims—see Shoemaker, 2011, for the need for this qualification) because the wrongness of these transgressions supervenes (inter alia) on the harm or distress they cause.

Because this is true, claiming that they are permitted elicits *imaginative resistance*, just as an author's attempt to stipulate that a concept applies to a fictional scenario despite also stipulating that the set of facts upon which that concept supervenes fails to obtain, elicits imaginative resistance. To that extent, there is nothing special about moral concepts and authority-independence: authority-independence arises in any domain in which concepts apply in virtue of facts other than the say-so of authorities, which is virtually everywhere (Levy, 2005). Given that victims’ distress is a central fact in virtue of which (some) moral concepts apply to wrongful actions, and psychopaths have difficulty with empathy, it is unsurprising that they have difficulties in using authority-independence as a criterion for morality. However, nothing prevents them from acquiring semantic knowledge of which actions are typically classified as morally wrong.

Suppose this interpretation of the performance of psychopaths is correct. In that case, they (or at least some significant proportion of them) have latched onto the pattern that normal people use in classifying rules. They are able to distinguish authority-dependent and independent rules reliably. Ought we to conclude that they have moral knowledge? Surely not: whatever the correct analysis of ‘knowledge’ might be, it is surely a necessary condition of an agent's knowing that p that the agent believes that p. Ability to track uses of p by others seems far short of sufficient.

Notice, by the way, how this argument differs from the traditional argument that psychopaths lack moral knowledge. The traditional argument turns on motivational judgment internalism, inferring that psychopaths lack moral knowledge from their failure to be motivated to act in accordance with the moral judgments they voice. In contrast, the argument just presented turns on the claim that in the most basic and straightforward sense of ‘believe’, psychopaths do not believe the moral judgments in question. Rather than turning on the alleged conceptual link between motivation and moral belief, this account turns on the empirical prediction that—given the right probes, and with incentives to impression management removed—psychopaths will deny that they see some kinds of prescriptions as binding on them.^3^

If the offered interpretation of the empirical evidence is correct, psychopaths lack certain central kinds of moral knowledge. However—again, on this interpretation of the evidence—there is clearly something the psychopath knows: s/he knows the rules. Further, the argument does not entail that s/he lacks moral knowledge altogether; it entails only that s/he lacks an understanding of moral wrongness insofar as it depends on causing distress. Might psychopaths be blameworthy in virtue of violating moral rules, or—for instance—acting viciously? It is at this point that we ought to turn to the content of the attitudes that are expressed in wrongdoing. In what follows, I will argue that regardless of whether the psychopath possess a kind of knowledge sufficient for satisfying the epistemic condition on moral responsibility, his/her actions nevertheless lack a kind of content that would justify (full-blown) blame.

## 4. Blame and the Content of Actions

### 4.1. Moral Blindness

Our reactive attitudes toward other agents should be strongly influenced by the moral content of their actions. An action has a content as a function of two factors: the agent's intentions in performing that action; and the facts of which the agent is (at least dispositionally) aware and which the agent takes to be potentially relevant to the action. Thus, the content of my action in typing these words is a function of my intention in doing so (to write a sentence, communicate an idea, complete a paper, or what have you) and the alternative actions that I am aware are available to me and that figure among the opportunity costs of my action. Our reactive attitudes are responses to the moral content of actions.

Following Arpaly (2002), we can grade the moral content of actions by reference to the reasons for which an agent acts. An action has the highest degree of moral content when the agent acts in response to moral reasons; it has a lower degree of moral content when the agent acts without regard to moral reasons of which s/he is nevertheless aware, and the lowest degree of moral content when the agent fails to register moral reasons at all, not even as considerations to be set aside or ignored. When the psychopath acts, I claim, his/her actions have a low degree of moral content, and therefore justify correspondingly weak reactive attitudes in response.

Perhaps the best way to drive this point home is by reference to the actions of an anthropologist in an alien community. Like the psychopath, our anthropologist (I stipulate) knows the rules of the community. But—again like the psychopath—s/he does not take the rules to be binding for him/her. Their force, for him/her, is due entirely to the fact that his/her subjects accept these rules as binding; were it not for that fact, s/he would not see him-/herself as having any reason to act in accordance with them (when s/he leaves the community, s/he ceases to attend to the rules). Suppose that s/he performs an action forbidden by the rules. What is the content of his/her action?

In answering this question, we need to note, and set aside, a difference between the anthropologist and the psychopath. Unlike the psychopath, the anthropologist believes that there are (welfare-referring) moral rules that apply to his/her behavior, and that these rules give him/her some reason to avoid violating the rules that his/her subjects accept. As we noted above, conventions are typically backed up by second-order moral rules: we typically have moral reasons to refrain from violating conventional rules. Thus, our anthropologist takes him-/herself to have a moral reason to avoid violation of the rules his/her subjects (wrongly, in his/her view) take to be moral rules: his/her doing so can be expected to cause them distress. Since nothing like this is true of the psychopath, illuminating the content of his/her actions via this analogy requires us to set aside this second-order moral content.

Further, there may be occasions on which our anthropologist violates a norm that obtains among his/her subjects because it is such a norm. S/he may be interested in how they respond to such violations. These cases should also be set aside. Psychopaths are distinguished by the extent to which they engage in instrumental, as well as reactive, violence; that is, they are uniquely willing to engage in harmful actions to further their goals, rather than in response to frustration or perceived challenges (Blair, Mitchell, & Blair, 2005). Cases in which psychopaths violate a norm just because it is a moral norm are likely to be vanishingly rare.

As we saw above, an action has the highest degree of moral content when it responds to moral reasons. Now, despite their deficits with regard to morality, psychopaths may sometimes respond to reasons that happen to be moral (though they will not conceive of them as moral reasons). As Arpaly (2002) herself stresses, an agent can be mistaken about the nature of the reasons to which s/he responds. However, the psychopath can neither respond to, nor even deliberately ignore or set aside, some central moral reasons. Consider the instrumental violence in which the psychopath is uniquely prone to engage. We typically blame agents for engaging in this kind of action because they are aware, or ought to be aware, that their actions cause a distinctive kind of distress. These acts are blameworthy because this fact—that the act causes a distinctive, and distinctively significant, kind of distress—is not given the weight it ought to be by the agent. But, while the psychopath may act knowing his/her actions cause distress and may deliberately ignore this fact, the content of his/her harmful action is different from, and a lower grade than, the content of the similar action of someone who understands moral distress. It is not qua distinctively moral reason against harm that s/he ignores the distress s/he causes.

The easiest way to see how this fact greatly reduces the moral content of his/her action is to swap the positions of the psychopath and the anthropologist. Suppose, that is, that the psychopath violates rules which are taken to be, but are not, moral rules (surely psychopathy is a disorder that can occur in any community). It seems clear that when s/he violates the local norms of a community which is fundamentally mistaken about aspects of morality—failing to help hunt down an escaped slave, for instance—s/he is to be excused because s/he neither responds to, nor even ignores, reasons that are genuinely moral. His/her actions therefore do not have a significant moral content. But when s/he violates genuinely moral rules, the situation is, from his/her point of view, identical: s/he takes him-/herself to have no more reason to guide his/her actions by the prevailing norms in the latter case than the former. His/her ignorance seems to excuse.

A caveat is in order. Above, I conceded two points: (1) that there are alternative routes to coming to know the content of rules against causing distress; and (2) morality is not exhausted by considerations concerning harm to others. The argument above is designed to show that (1) does not justify condemnatory reactive attitudes toward the psychopath. The fact that one violates rules that one knows are regarded as especially binding does not justify condemnation, because the moral content of such actions may be slight. Even though psychopaths may regularly come to know what the rules proscribe, their violations of these rules do not have the kind of content that justifies full-blown blame. The argument does not, however, show that blame might not be warranted via some pathway turning on the aspects of morality adverted to by (2), those aspects which do not depend on considerations of harm to others (recall the finding by Aharoni et al. that psychopaths are responsive to some of the so-called “foundations” of morality).^4^ For all that has been said here, psychopaths may be capable of grasping the virtues (say), in their distinctively moral meaning. To that extent, psychopaths may be justifiable targets of moral condemnation.

However, I take it as obvious that the central reason for condemning major harms to non-consenting others turns on the way in which these harms cause distress, not on the ways in which they may express vices, even if (what is not obvious) we can understand these vices without reference to the harms. This is relatively uncontroversial, even among virtue ethicists. The psychopath may deserve some degree of condemnation on the basis of those facets of morality s/he does grasp, if indeed there are any, but the degree will be relatively small because most of the work, in the normal case, is done by those aspects to which s/he is blind. This fact will entail that the moral content of his/her actions will usually be slight.

### 4.2. Mental Time Travel

I turn now to a second deficit from which some psychopaths suffer: a deficit with regard to mental time travel (MTT). I shall argue that this deficit also plays a role in reducing the moral content of their actions. Psychopaths cannot intend the sort of harm to which only persons can be subject. Thus, they can intend to cause distress—just as non-psychopaths can intend to cause distress to those non-human animals that are not persons—but not these distinctive harms.

MTT is the ability to project oneself into the future or the past: to recall, in a distinctively first-person manner, past episodes and to simulate possible future scenarios in which one is personally engaged (Suddendorf & Corballis, 2008). Psychologists call the kind of recall involved in MTT to the past episodic memory (Tulving, 2002). To recall in this manner is to recall an event as one in which one was oneself engaged. We recall not merely that the event occurred, but also just how it occurred; this may involve replaying the episode from the perspective originally experienced, or it may involve replaying the experience as if we were observing it from outside. Episodic memory for an event requires that we were actually involved in that event (unlike semantic memory: I may have semantic memory for events in which I was not involved) and the right kind of causal connection between the event and the recollection. Mental time travel to the future—prospection, as it is sometimes called—is the future-oriented version of the same phenomenon.

Mental time travel may have evolved for the control of behavior (Boyer, 2008). For most of evolutionary history, animals had to engage in relatively little long-term planning. Some planning was necessary (predators needed to engage in strategies that would allow them to come within striking range of prey; herbivores needed to seek foraging opportunities, and so on) but most plans had payoffs delivered in, at most, hours. Human beings alone plan for the much longer term. Motivating an animal that is evolved to respond to immediate incentives to plan for the long-term is a difficult task, one which our well-documented failures at self-control attests we have not completely solved. However, our obvious success at pursuing education, at writing books and articles, at composing music, and so on, shows that we have managed to overcome bias toward the immediate sufficiently well to engage in long-term projects. Mental time travel may help to enable these achievements: the vivid imagining of future success may allow us now to experience some of the emotions that we expect to experience at the completion of a project, thereby mitigating the problem of temporal discounting of future goods.

MTT to the past involves recall; prospection probably utilizes much the same network involved in recollection (Gerrans & Kennett, 2010). Thus, an impairment to memory systems may entail an impairment to MTT in both its forms. It is therefore important to note that psychopaths suffer from problems with memory. Christianson et al. (1996) and Dolan and Fullam (2005) both found that psychopaths had worse recall for emotional materials than controls. However, this might not indicate a memory problem per se; rather, it might indicate that due to their affective impairments, psychopaths fail to benefit from the boost to recall that normals receive with regard to emotional stimuli. On this hypothesis, the memory impairment would be due to factors outside the domain of memory per se.

However, even if emotional impairments explain some or all of the deficits exhibited on the recall of emotional materials, there is plentiful independent evidence that some psychopaths suffer from deficits with regard to mental time travel (Kennett & Matthews, 2009). Psychopaths engage in ordinary short-term planning; their use of instrumental violence is itself evidence of this fact, but they are notoriously bad at achieving their goals. Impulsivity and a failure to plan ahead is a diagnostic criterion for antisocial personality disorder, the DSM (IV-R) category that is closest to psychopathy. Psychopaths lack realistic goals, and have no plans at all for how to achieve the goals they do have.

One incarcerated psychopath, for example, told interviewers that he intended to become a chef, a surgeon, a pilot, and an architect (Hare, 1999). Since he possessed qualifications for none of these professions, these ideas seem more akin to fantasies than to genuine goals, insofar as goals are ends toward which people take themselves to be working. McIlwain (2010) understands this behavior as the consequence of an inability to transcend the present moment in imagination, in the kind of engaged manner required for MTT. Consistent with the hypothesis that MTT enables self-control, she adds, psychopaths appear unable to restrain even fleeting impulses.

Petrican and Burris (2011) provide further evidence that psychopaths feel “stuck in the present.” They studied the construct they call temporal inefficacy (TI), which they define as discontentment with a perceived lack of control over time experienced as linear and inescapable. Higher TI scores, as measured using a self-report scale, correlate with a focus on the present. Further, TI correlates with lower scores on an autobiographical recall task and a greater willingness to subordinate one's intimate partner's wellbeing to one's own. TI is also positively correlated with higher scores on a scale drawn from the Hare Self-Report Psychopathy-III scale, focusing on a callous, self-serving, and manipulative style. We should be hesitant on placing too much weight on this evidence, given that Petrican and Burris did not focus on psychopaths directly. However, the study provides some degree of convergent evidence: together with all the other data, this evidence suggests that psychopaths are likely to do worse on autobiographical recall and feel “stuck in the present” to a greater degree than controls. Both of these characteristics indicate a problem with mental time travel.

This impairment, I suggest, greatly reduces the moral content of psychopaths’ actions that intentionally harm persons. Personhood depends on the capacity for conceiving oneself as a persisting being, with plans and projects of one's own; the distinctive harm involved in killing a person, as opposed to a non-person, arises from interrupting these plans and projects. It is this capacity for personhood that gives someone an interest in continued life (Singer, 1993; McMahan, 2002). Lacking the capacities required for personhood, most non-human animals have an interest in avoiding suffering but not in continuing to live. It is wrong to harm non-persons, but killing them is very much less wrong than killing persons. It may not be (directly) wrong at all.

The same facts that entail that killing a person is much more morally significant than killing a non-person also entail that harming a person is at least typically more morally significant than harming a non-person. A person's capacity for pursuing plans and projects is the capacity for autonomous action: for action that imposes a shape on that person's life. At their most serious, these actions aim at the person's conception of the good: their idea of what values a life should instantiate. Causing harm to persons interferes with the exercise of this capacity and therefore threatens his/her autonomy.

These facts affect the content of actions that are intended to harm or kill persons. Understanding that someone is a person and harming or killing him/her despite this knowledge entails an action that has heavily freighted moral content. The person who kills another acts in defiance of one of the most serious moral reasons of all; the person who harms another acts in defiance of the knowledge that they thereby violate the autonomy of that other. The moral content of an act of harming or killing a non-person, or a person wrongly but non-cupably taken to be a non-person, is much lower, because the agent who acts in this way does not act in defiance of these special sorts of reasons.

The capacities for being a person overlap very extensively with, and may even be identical to, the capacities that underlie MTT. Planning for the future involves imaginative projection; it requires that we understand the actions we undertake *now* as getting their point from a goal that may not be realized for weeks, months, or (often enough) years. This requires prospection. It also requires that we identify with our past stages and see them as engaged in a project which we share and continue. It follows that an inability to engage in full-blown MTT entails an impairment in personhood. It also very probably entails an impaired ability to grasp what it is to be a person, with plans and projects.

Some of the callousness of psychopaths may arise from the fact that they cannot understand the impairment to autonomy their actions cause, because they cannot understand what it is to be fully autonomous. Failure of empathy is of course characteristic of the psychopath, and certainly, a great deal of their deficits in this area can be explained by a failure to grasp the emotional states of others. Consider, for instance, the mystification expressed by a psychopathic rapist with regard to his victims: “they are frightened, right? But, you see, I don't really understand it. I've been scared myself, and it wasn't unpleasant” (Hare, 1999, p. 44).

Crucially, however, some of the psychopaths’ failures to understand the distress of victims do not seem due to a failure to understand the emotions of their victims; rather, they seem due to a failure to appreciate that others have lives; ongoing plans and projects through which persons impose their conceptions of the good on themselves. It is not lack of empathy for his victim's mental states that leads one psychopath to say of the man he murdered that he had “learned a hard lesson about life” (Hare, 1999, p. 41), nor another who conned and robbed vulnerable elderly women to conclude that the women “got their money's worth” from his attentions (Hare, 1999, p. 30). One psychopath candidly confesses that he felt no worse about the people he harmed than about squashing a bug (Hare, 1999, p. 33). From his perspective, there is little difference: neither bug nor human is understood as an autonomous being.^5^

The evidence powerfully suggests that psychopaths have a significantly attenuated conception of what it is to be a person. Correlatively, they cannot intend the kind of harms that can befall only persons. The content of their actions does not include, even as a consideration to be set aside or ignored, the infringement of the autonomy of their victims, i.e., the manner in which being harmed interferes with the victim's plans and projects. Nor can they intend the kind of harm in killing that befalls only persons, i.e., the harming consisting in the permanent cessation of one's future-oriented plans and projects. For this reason, the moral content of psychopaths’ actions is likely to be significantly smaller than the moral content of similar acts of non-psychopathic offenders.^6^ Our reactive attitudes ought to be similarly attenuated; they deserve a lower degree of condemnation for their actions.

It is worth emphasizing, however, that the deficits with regard to MTT—and therefore, any extenuation these deficits warrant—may be restricted to lower-functioning psychopaths. Higher-Functioning psychopaths—so-called “snakes in suits” (Babiak & Hare, 2006)—seem to be able successfully to pursue plans over time; they therefore appear to have fewer problems with MTT (alternatively, they may have found a way to compensate for these deficits; whether this compensatory route would also entail ineligibility for extenuation is currently unknown). Though this fact restricts the scope of any extenuation due to psychopaths, in practice, this restriction may be relatively unimportant, inasmuch as it is lower-functioning psychopaths who are more likely to commit violent crimes like murder and assault. This caveat ought to be borne in mind, however. In concluding, I will mention other limitations of the arguments presented here.

## 5. Conclusions and Caveats

I suggest that the considerations advanced, with regard to the psychopath's understanding of morality and with regard to their understanding of personhood, should significantly reduce the amount of blame directed at psychopaths. Their actions do not have the kind of moral content that would justify full-blown condemnation. Coupled with the independent considerations from their apparent—and apparently excusing—lack of moral knowledge, we have a powerful case for excusing them, or at least very significantly mitigating moral condemnation directed at them.

Now for the caveats. First, important elements in my argument—one crucial for the entire argument, one for the second argument turning on MTT—have gone undefended. Rather than arguing for the claim that reactive attitudes should be a function, in part, of the moral content of actions, I have simply stipulated it, and referred the reader elsewhere for defenses of the claim. Similarly, I have not defended the account of personhood upon which the argument from MTT turns; again, I have referred the reader elsewhere. These claims are plausible, but nothing said here forces the reader to agree. Nevertheless, insofar as these claims are widely accepted, the arguments put forward here should be accepted by many, and taken seriously by everyone.

This first caveat concerns a possible weakness in the argument. The second one concerns its scope. There is an ongoing debate whether psychopathy is a taxon—a discrete class—or whether, instead, psychopaths exist on a continuum (Harris, Rice, & Quinsey, 1994; Edens, Marcus, Lilienfeld, & Poythress, 2006). If psychopathy should be understood as existing along a continuum, or even if the traits on which my argument turns—the capacity to understand morality and the capacity for MTT—exist along a continuum in psychopaths, different psychopaths will perform actions with different degrees of moral content, and therefore might be due differing amounts of blame. As already mentioned, some psychopaths seem capable of a relatively high degree of planning and impulse control, which seem in turn to depend on some capacity for MTT. Correlatively, these psychopaths might be due a higher degree of moral condemnation for their actions. Rather than concluding that their impairments with regard to MTT mitigate psychopaths’ moral responsibility, then, we should say that to the extent that their impairment with MTT reduces the moral content of their actions, their moral responsibility is similarly reduced. Similar remarks apply to the moral content of their actions insofar as this is a function of the capacity to understand harms.

Despite these caveats, the considerations advanced here constitute a powerful argument to reduce the amount of blame due to many, perhaps very many, psychopaths. It might even play a role in reducing the moral responsibility of some of them to zero.

## Notes

[1] As a referee for this journal points out, this interpretation is bolstered by the fact that psychopaths were also significantly less likely to justify why a moral transgression is wrong by reference to victims’ welfare than were controls (Blair, 1995, p. 18).

[2] It should be noted, however, that the subjects in Glenn et al. (2009) were not psychopaths. Rather, the study examined psychopathic traits (identified by self-report) in a group of non-psychopaths.

[3] This is an empirical claim, and therefore, a potentially testable one. However, identifying appropriate probes is no trivial task. The hypothesis predicts that there are actions that normal controls will identify as moral transgressions but psychopaths will fail to identify as transgressions at all. I suggest that the right probes will involve novel actions that cause subtle kinds of psychological harms to persons (perhaps by failing to attend to their remarks or by failing to make them feel comfortable in social situations). Given that psychopaths have particular difficulty in understanding the badness of such harms, when the wrongness of a transgression is both novel (so that the subjects will not have had the opportunity to observe others reacting to these transgressions) and involves these kinds of harms, these actions seem the best bet to generate the predicted divergence between psychopaths and controls. The harms might be buried in vignettes, short descriptions of interactions. I predict that normal controls will have no trouble in identifying the harms involved but psychopaths will struggle.

[4] An anonymous referee suggests an alternative route whereby psychopaths might be held to be morally responsible for moral harms despite the deficits they exhibit: if their disgust responses are normal, they will feel disgust at the effects of violations of the body (bodily fluids are paradigm elicitors of disgust). They will then be able to classify bodily violations as moral transgressions, via the sanctity/degradation moral foundation identified by Haidt (2012). Even if Haidt is correct in identifying six independent foundations of morality, each of which has a right to be counted as genuinely and significantly moral (which I doubt), however, it is far from clear that all the moral foundations are apt for providing an independent foundation for blame and other reactive attitudes. The question is an interesting one that deserves a full exploration; unfortunately, I cannot enter into it here.

[5] An anonymous referee for this journal notes that were someone suffering from the delusion that s/he was actually killing bugs and not people, s/he would be found not guilty by reason of insanity. To the extent to which the situation, as the psychopath perceives it, is relevantly identical to the situation confronting the delusional defender, it would seem that they should have access to the same defense. Any degree of genuine culpability would seem to depend on (a) the extent to which they have epistemic access to moral reasons (if psychopathy comes in degrees, as seems likely, this may vary from case to case), and (b) the degree to which they have epistemic access to the kinds of moral facts that can underwrite blame via other routes.

[6] The claim made here is that because psychopaths lack the concept of personhood, they cannot intend a harm to a person, and that this fact reduces the moral content of their intentional actions that harm persons. As a referee for this journal points out, however, psychopaths’ deficits with MTT may entail that they are not themselves persons, and for this reason not fit subjects of the reactive attitudes. The links between personhood and aptness for moral responsibility are subtle and intricate, and deserve a separate discussion, so I do not enter into these waters here.

## References

[R1] Aharoni E., Sinnott-Armstrong W., Kiehl K.A. (2012). Can psychopathic offenders discern moral wrongs? A new look at the moral/conventional distinction. Journal of Abnormal Psychology.

[R2] Arpaly N. (2002). Unprincipled virtue: An inquiry into moral agency.

[R3] Babiak P., Hare R.D. (2006). Snakes in suits: When psychopaths go to work.

[R4] Blair R.J.R. (1995). A cognitive development approach to morality: Investigating the psychopath. Cognition.

[R5] Blair R.J.R. (1997). Moral reasoning in the child with psychopathic tendencies. Personality and Individual Differences.

[R6] Blair R.J.R. (2007). What emotional responding is to blame it might not be to responsibility. Philosophy, Psychiatry, & Psychology.

[R7] Blair J., Mitchell D., Blair K. (2005). The psychopath: Emotion and the brain.

[R8] Boyer P. (2008). Evolutionary economics of mental time-travel?. Trends in Cognitive Sciences.

[R9] Brink D. (1989). Moral realism and the foundations of ethics.

[R10] Christianson S., Forth A.E., Hare R.D., Strachan C., Lidberg L., Thorell L. (1996). Remembering details of emotional events: A comparison between psychopathic and nonpsychopathic offenders. Personality and Individual Differences.

[R11] Dolan M., Fullam R. (2005). Memory for emotional events in violent offenders with antisocial personality disorder. Personality and Individual Differences.

[R12] Dolan M., Fullam R. (2010). Moral/conventional transgression distinction and psychopathy in conduct disordered adolescent offenders. Personality and Individual Differences.

[R13] Edens J.F., Marcus D.K., Lilienfeld S.O., Poythress N.G. (2006). Psychopathic, not psychopath: Taxometric evidence for the dimensional structure of psychopathy. Journal of Abnormal Psychology.

[R14] Gerrans P., Kennett J. (2010). Neurosentimentalism and moral agency. Mind.

[R15] Glenn A.L., Iyer R., Graham J., Koleva S., Haidt J. (2009). Are all types of morality compromised in psychopathy?. Journal of Personality Disorders.

[R16] Haidt J. (2012). The righteous mind: Why good people are divided by politics and religion.

[R17] Haidt J., Joseph C. (2004). Intuitive ethics: How innately prepared intuitions generate culturally variable virtues. Daedalus.

[R18] Haji I., Malatesti L., McMillan J. (2010). The inauthentic evaluative schemes of psychopaths and culpability. Responsibility and psychopathy: Interfacing law, psychiatry and philosophy.

[R19] Hare R.D. (1999). Without conscience: The disturbing world of the psychopaths among us.

[R20] Harris G.T., Rice M.E., Quinsey V.L. (1994). Psychopathy as a taxon: Evidence that psychopaths are a discrete class. Journal of Clinical Psychology.

[R21] Kennett J., Matthews S., Broome M., Bortolotti L. (2009). Mental time travel, agency and responsibility. Psychiatry as cognitive neuroscience: Philosophical perspectives.

[R22] Levy N. (2005). Imaginative resistance and the moral/conventional distinction. Philosophical Psychology.

[R23] Levy N. (2007). The responsibility of the psychopath revisited. Philosophy, Psychiatry & Psychology.

[R24] Levy N., Malatesti L., McMillan J. (2010). Psychopathy, responsibility and the moral/conventional distinction. Responsibility and psychopathy: Interfacing law, psychiatry and philosophy.

[R25] McIlwain D. (2010). Living strangely in time: Emotions, masks and morals in psychopathically-inclined people. European Journal of Analytic Philosophy.

[R26] McMahan J. (2002). The ethics of killing: Problems at the margins of life.

[R27] Nucci L.P., Nucci L.P. (1989). Challenging conventional wisdom about morality: The domain approach to values education. Moral development and character education: A dialogue.

[R28] Petrican R., Burris C.T. (2011). The infernal now: Linking temporal inefficacy to cognitive ability and social adjustment. Canadian Journal of Behavioural Science.

[R29] Scanlon T.M. (1998). What we owe to each other.

[R30] Scanlon T.M. (2008). Moral dimensions: Permissibility, meaning, blame.

[R31] Shoemaker D.W. (2011). Psychopathy, responsibility, and the moral/conventional distinction. Southern Journal of Philosophy.

[R32] Singer P. (1993). Practical ethics.

[R33] Suddendorf T., Corballis M.C. (2008). The evolution of foresight: What is mental time travel and is it unique to humans?. Behavioral and Brain Sciences.

[R34] Tulving E. (2002). Episodic memory: From mind to brain. Annual Review of Psychology.

[R35] Turiel E. (1977). Distinct conceptual and developmental domains: Social convention and morality. Nebraska Symposium on Motivation.

[R36] Turiel E. (1983). The development of social knowledge: Morality and convention.

[R37] Vargas M., Nichols S. (2007). Psychopaths and moral knowledge. Philosophy, Psychiatry, & Psychology.

